# Differences in upper body posture between patients with lumbar spine syndrome and healthy individuals under the consideration of sex, age and BMI

**DOI:** 10.1186/s12995-024-00405-w

**Published:** 2024-02-14

**Authors:** Fabian Holzgreve, Celine Nazzal, Rasem Nazzal, Rejane Golbach, David A. Groneberg, Christian Maurer-Grubinger, Eileen M. Wanke, Daniela Ohlendorf

**Affiliations:** 1 Institute of Occupational Medicine, Social Medicine and Environmental Medicine, Goethe University Frankfurt, Theodor-Stern-Kai 7, Building 9a, 60596 Frankfurt am Main, Germany; 2 Physiotherapy practice, Dr. Rasem Nazzal, Frankfurt, Germany; 3 Institute for Biostatistics and Mathematical Modeling, Center of Health Sciences, Goethe University Frankfurt, Frankfurt am Main, Germany

**Keywords:** Low back pain, Video raster stereography, Lumbar lordotic angle, Kyphosis angle, Lordosis angle, Regression analyses, Kinematics, Standing posture

## Abstract

**Background:**

Work-related forced postures, such as prolonged standing work, can lead to complaints in the lower back. Current research suggests that there is increased evidence of associations between patients with low back pain (LBP) and reduced lordosis in the lumbar spine and generally less spinal tilt in the sagittal plane. The aim of this study is to extend the influence of LBP to other parameters of upper body posture in standing, taking into account the rotational and frontal planes.

**Methods:**

The study included a no-LBP group (418 males, 412 females, aged 21–65 years) and an LBP group (138 subjects: 80 females, 58 males, aged 18–86 years) with medically diagnosed lumbar spine syndrome (LSS). The “ABW BodyMapper” back scanner from ABW GmbH in Germany was used for posture assessment using video raster stereography. Statistical analyses employed two-sample t-tests or Wilcoxon-Mann-Whitney-U tests to assess the relationship between the LBP/no-LBP groups and back posture parameters. Linear and logarithmic regressions were used with independent variables including group, sex, height, weight and body mass index (BMI). Significance level: α = 0.05 (95% confidence).

**Results:**

The regression analysis showed that sagittal parameters of the spine (sagittal trunk decline, thoracic and lumbar bending angle, kyphosis and lordosis angles) depend primarily on sex, age, BMI, height and/or weight but not on group membership (LBP/no-LBP). In the shoulder region, a significant dependency between group membership and scapular rotation was found. In the pelvic region, there were only significant dependencies in the transverse plane, particularly between pelvic torsion and BMI, weight, height and between pelvic rotation and group membership, age and sex.

**Conclusion:**

No difference between the patients and healthy controls were found. In addition, sex appears to be the main influencing factor for upper body posture. Other influencing factors such as BMI, height or weight also seem to have a significant influence on upper body posture more frequently than group affiliation.

## Introduction

Work-related musculoskeletal disorders are an increasing challenge for employees and employers [1]. A common complaint in an occupational context is low back pain (LBP) which imposes a significant economic burden in the United States, exceeding $100 billion annually. About two-thirds of these costs are indirect, arising from lost wages and reduced productivity due to the consequences of low-back pain [2]. Low back pain is caused by the fact that the lumbar spine is particularly frequently affected, as it is exposed to an increased risk due to various occupational activities, such as standing work or forced postures [3, 4]. The consequences range from temporary discomfort to long-term incapacity to work. The global prevalence of lower back pain reached a substantial 619 million individuals in the year 2020. This research underscores the urgent need for targeted studies and interventions to address the escalating burden of lower back pain on a global scale and tailor strategies to regions with the highest prevalence [5].

Any pain in the lumbar spine area that does not represent a clearly defined disease subsumes the lumbar spine syndrome (LSS). It involves non-specific acute, chronic, structural, degenerative, traumatic and inflammatory processes that can lead to permanent poor alignment of the spine and poor posture [6]. Impairment in the lower back can, for example, be a kyphosis or lordosis angle (radiographic norm: 20° to 60°, according to Cobb [6]) that deviates further from the radiographic norm (25–40° according to Cobb [6]) as well as a hyperkyphosis or a scoliosis [6, 7]. Furthermore, Scheuermann’s disease, prolapse, vertebral body hernias or deformities, osteoporosis, lumbago, rheumatoid arthritis or spondylolisthesis can also contribute to the development of LSS; here, these pathologies are usually of multifactorial etiology [7–11].

Taking into account the heterogeneous study situation, Chun et al. [12] came to the conclusion that people with LBP tend to have a smaller lumbar lordotic angle (LLA) compared to age-matched healthy individuals. Here, X-ray, magnetic resonance imaging (MRI) and computed tomography (CT) were used to determine the Cobb angle. From a biomechanical point of view, the authors suspect the loss of lordosis as a possible cause of lumbar spine pain. Chaléat-Valayer et al. [13] also investigated whether the sagittal alignment of the spine and pelvis is more pronounced in chronic low back pain. Their evaluation of radiographs when standing (sagittal spino-pelvic alignment) revealed a greater proportion of chronic LBP patients with low sacral slope, low lumbar lordosis and low pelvic incidence. This suggests a correlation between this specific pattern and the presence of chronic LBP, although the significant mean values differ by only 3.7° between subjects with LBP and healthy subjects, at most. Even in asymptomatic individuals, the sagittal alignment of the spine and pelvis is subject to natural variation. They concluded that, clinically, there must be several factors that can trigger lumbar pain. Thus, this could explain the possible inconsistency of data interpretation across LBP studies and why the relationship between sagittal alignment and LBP is still not yet understood. Laird et al. [14] compared the biomechanical aspects of lumbo-pelvic movement using skin surface measurement techniques to measure the lumbo-pelvic posture or movement in people with lumbar spine complaints compared to those without. They found a reduced lumbar range of motion (ROM) with slower movements and reduced proprioception in LBP patients. Nonetheless, they could not prove whether these deficits already existed before the onset of lumbar spine pain.

In addition, other factors influencing LBP, such as gender, body mass index (BMI) and age, were taken into account in various analyses. Radiographic comparisons of 148 lumbar spine patients and 148 control subjects showed that female gender, higher BMI, smoking and blue-collar jobs were associated with a higher risk of non-specific lumbar spine pain, with lumbar lordosis, sacral tilt and pelvic tilt all being greater in lumbar spine patients [15]. Using a durometer inclinometer, Król et al. [16] found that people with and without pain differed significantly in terms of anterior pelvic tilt (higher in people without LBP). The risk of LBP increased with age in the study group. Further radiographic analysis of sagittal plane alignment and balance in standing volunteers and patients with low back pain (matching data for age, gender and height) showed significantly less lordosis in patients compared to a control group and similar thoracic kyphosis [17]. Korovessis et al. [18] found most dependencies to be only from the sixth decade onwards (radiographic images): the thoracic kyphosis of the control group increased, while the sacral inclination decreased with increasing age; this was less pronounced in LBP patients. However, the LBP pain group had a more pronounced thoracic kyphosis and less pronounced lordosis. In contrast, Abrisham et al. [19] found that the age of the subjects had no influence on the kyphosis and lordosis angles (EOS: ø lordosis: 32.42°, ø kyphosis: 43.55°) and when over 40 years of age, the kyphosis angle values of men were found to be greater than those of women.

The presented studies show that thoracic changes due to LBP or frontal and transverse influences are less researched in this regard. Furthermore, the focus was less on the entire pelvic area including rotational or transverse changes. Such relationships are easy to capture with non-invasive video raster stereography since the dorsal upper body posture from C7 to the rima ani are captured in three dimensions. Therefore, the aim of the present analysis was to investigate differences in upper body posture between patients with medically diagnosed LSS and healthy individuals under the consideration of sex, age and BMI.

## Methods

### Subjects

The no-LBP group included 418 males and 412 females between the ages of 21 and 65 years. The no low back pain (no-LBP) data have already been published to generate norm values for the upper body posture and were used again for this analysis [20].

All participants considered themselves to be in good health at the time of the measurements, meaning they reported no orthopaedic or neurological issues and no history of musculoskeletal system injury or surgery. For female participants, it was ensured that they were not pregnant at the time of measurement or, if they had given birth, that it had been at least six months were found to be not. Participants were gathered from the Frankfurt am Main local area through a combination of flyers and word of mouth. For the LBP group, 138 (80f/58m) subjects aged between 18 and 86 years were studied. The inclusion criteria for subjects in the LBP group were medically diagnosed LSS and a minimum age of 18 years for all participants. All included subjects were patients of a physiotherapy practice and had been prescribed physiotherapeutic treatment by an orthopaedist following a medical diagnosis of LSS. Furthermore, the low back pain was not quantified more precisely. All participants were measured before starting the therapy. They were all referred by an orthopaedist for physiotherapy treatment following the diagnosis of LSS. The medical diagnosis was called LSS and was made by the orthopaedist. A more detailed description of the diagnosis was not given.

Exclusion criteria comprised acute complaints in other areas of the body and neurological known complaints that would affect posture. Other current therapy for the complaints was also excluded.

An approved ethics application was submitted for the conduct of the study (Ethics No.: 20/17). Its specifications refer to the ethical principles for medical research involving human subjects that are set out in the current version of the Declaration of Helsinki from 2013.

### Three-dimensional back scanner

**Fig. 1:**
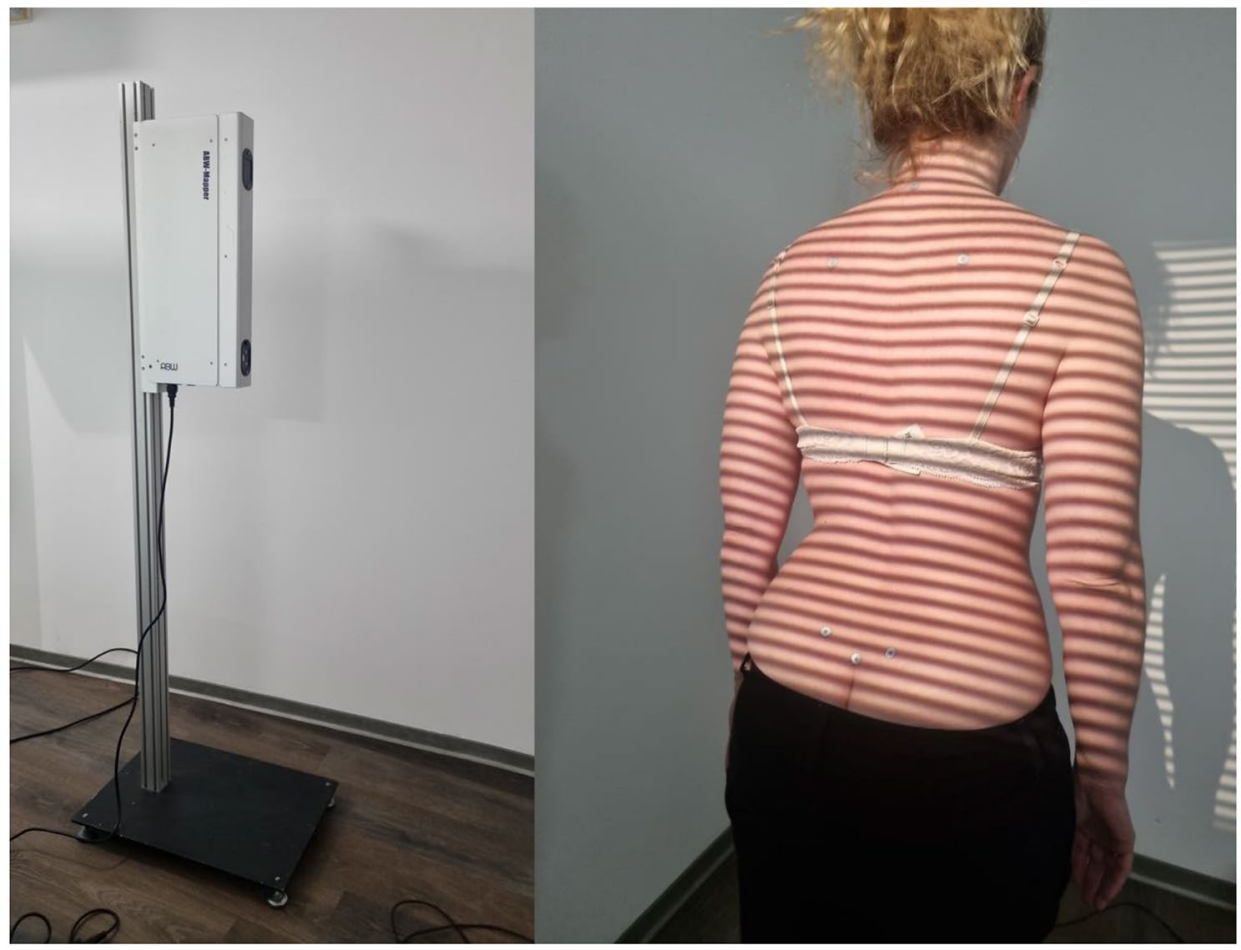
Three-dimensional back scanner (left) and marker placement (right). Markers are placed on the following landmarks: vertebra prominens (7th cervical vertebra), scapular angle right, scapular angle left, dimple right, dimple left, sacrum point (beginning of the rima ani)

The “ABW BodyMapper” back scanner from ABW GmbH in Frickenhausen, Germany, was used to record posture in the upper body region, including the spine, shoulders and pelvis (Fig. 1). The principle used for the acquisition of data is called video raster stereography; this allows the creation of a three-dimensional image without the need for any contact. The scanner creates 50 frames per second during an image capture; the maximum frame rate is 50 frames per second. A depth resolution of 1/100 mm is specified by the manufacturer, with the measurement error specified as being less than 1 mm and the repeatability with repeated measurements as being more accurate than 0.5 mm (ABW GmbH company; Frickenhausen, Germany). The physical principle behind video raster stereography is called triangulation. In this process, the projector, camera and the point to be measured on the body surface form a triangle. The base length of this triangle (the distance between the projector and the camera) and the angles α and β between the emitted and recorded light beams are known. The light projector projects a grid of lines onto the patient’s back that is recorded by a camera. Software can create a three-dimensional image based on the curvatures of the lines. The curvature of the surface then allows reconstruction of the spatial treatment following the diagnosis of LSS.

### Measurement protocol

All subjects removed their shoes and outer clothing. Women were allowed to keep wearing their bra as long as it did not cover the important fixed points on the back. Jewellery and long earrings were also removed to eliminate potential sources of error in this light-sensitive method of back scanning. Subsequently, six self-adhesive and light-reflective markers with a diameter of 1 cm were applied to the fixed points on the patients’ backs (Fig. 1). The six fixed points were as follows:

1. VP: vertebra prominens (7th cervical vertebra).
2. AISR: scapular angle right.
3. AISL: scapular angle left.
4. DR: dimple right.
5. DL: dimple left.
6. SP: sacrum point (beginning of the rima ani).

Patients were then asked to stand on a template located 90 cm from the back scanner. The template served as a guide for foot alignment. A slightly outward angled foot position of 6° was aimed to represent the habitual position [21]. In addition, the subjects were asked to adopt a relaxed posture with their arms hanging relaxed beside the body. The gaze of the person being measured treatment following the diagnosis of LSS straight ahead and approximately at head level. It was important that the room was darkened to avoid any external light interference.

Furthermore, patients were not allowed to move during the measurement as this could lead to image distortion. The measurement was performed five times and the values were subsequently averaged for further statistical evaluation.

The parameters listed below and the outcomes of the back scan analyses were included in this study, while constitutional parameters, such as trunk length or pelvis/shoulder distance, were excluded:

- **Sagittal trunk decline** (inclination of the trunk length, D, marked line from the perpendicular to the sagittal plane) (°).
- **Frontal trunk decline** (inclination of the trunk length, D, marked line from the perpendicular to the frontal plane) (°).
- **Axis decline** (deviation of the line of the area marked by the trunk length, D, line of the 90° rotated distance between posterior superior iliac spine (PSIS) left and PSIS right) (°).
- **Thoracic bending angle** (deviation of the distance C7– kyphosis apex from the perpendicular) (°).
- **Kyphosis angle** (angle between the upper turning point at C7 and the thoracolumbar inflection point) (°).
- **Lumbar bending angle** (deviation of the distance kyphosis apex– lordosis apex from the perpendicular) (°).
- **Lordosis angle** (angle between the lower inflection point at the centre of the PSIS marker and the thoracolumbar turning point) (°).
- **Standard deviation of lateral deviation** (root mean squared deviation of the median line of the distance C7– centre of the PSIS marker) (mm).
- **Standard deviation of rotation** (root mean square deviation of surface rotation of the median line (torsion of the spinous processes of the spine)) (°).
- **Scapula rotation** (rotation of the distance AISL — AISR in the transversal plane) (°).
- **Scapula angle left** (angle of the compensation line applied to the shoulders to the horizontal. The centre of the compensation line is specified vertically above AISL) (°).
- **Scapula angle right** (angle of the compensation line applied to the shoulders to the horizontal. The centre of the compensation line is specified vertically above AISR) (°).
- **Pelvis torsion** (PSIS L - PSIS R twist around the transverse axis calculated from the mutual twisting of the surface normal on the two PSIS) (°).
- **Pelvis rotation** (rotation of the distance PSIS L– PSIS R in the transversal plane) (°).

### Statistical analysis

Statistics were conducted using SPSS Statistics 29 (IBM Deutschland GmbH, Ehningen, Germany) and Excel 2016 (Microsoft Corporation, Redmond, WA, USA). Initially, all data were tested for normal distribution using the Kolmogoroff-Smirnoff-Lilliefors test. Since the sample size was large, thus making the probability of significant differences more likely, the distribution of the data was also controlled by looking at the histogram and Q-Q diagrams. Descriptive statistics were applied using the mean and SD since the subject’s data were normally distributed. For inferential statistics, different methods were applied. In order to measure the relationship between the groups (LBP/no-LBP) and the dependent variables (back posture parameters), either the two-sample t-test or the Wilcoxon-Mann-Whitney-U test was used, depending on the distribution of the dependent variable. Furthermore, multivariate and logistic regressions using the independent values group (LBP/no-LBP), sex, height, weight and BMI dependency on data distribution were applied. Data with no normal distribution were firstly transformed using the natural logarithm. If the distribution became normal, then we calculated a linear regression with the transformed data. However, if the data remained non-normally distributed, then we dichotomised the data and applied a logarithmic regression. Outliers were dealt with as follows: first, the leverage value for each subject was determined. If the leverage effects from potential outliers in individual analyses were smaller than 2*mean of the leverage effects from all points, then the potential outliers were excluded from the respective analysis. The significance level was set to α = 0.05.

## Results

The subject data showed mostly minor differences between the groups (Table 1). However, the subjects of the no-LBP group were 3.47 years younger (*p* = 0.014; -5.66 - -1.27) than the subjects of the LBP group. Furthermore, sex was not evenly distributed. While sex was very homogeneously distributed (412f/418m) in the no-LBP group, the proportion of females in the LBP group was larger (80f/58m) (chi^2^-test: *p* = 0.085).

**Table 1:** Subject data of the no-LBP and LBP groups

	no-LBP group			LBP group			
	all (*n* = 830)	females (*n* = 412)	males (*n* = 418)	all (*n* = 138)	females (*n* = 80)	males (*n* = 58)	
Age ± SD (years)*	40.46 ± 11.46	39.85 ± 11.61	41.06 ± 11.29	43.93 ± 15.63	43.9 ± 14.9	43.4 ± 16.80	
BMI ± SD (kg/m²)	25.04 ± 4.34	23.91 ± 4.66	26.16 ± 3.68	25.0 ± 4.34	24.1 ± 4.80	26.0 ± 3.30	
Weight ± SD (kg)	76.01 ± 16.38	66.59 ± 12.89	85.41 ± 13.91	75.15 ± 16.07	67.4 ± 14.40	85.2 ± 12.50	
Height ± SD (m)	1.74 ± 0.01	1.67 ± 0.06	1.80 ± 0.07	1.73 ± 0.01	1.7 ± 0.06	1.8 ± 0.07	

Asterisks indicate significant differences between the groups

### Inferential statistics

The comparison of multiple upper body posture parameters in subjects with and without LBP showed significant differences in the frontal plane (frontal trunk decline, *p* = 0.001), meaning that the subjects with LBP tended to lean more to the left (Table 2). Further differences were seen in the scapular posture. Here, the right scapular of the no-LBP group was less balanced, resulting in a more dorsal rotated scapular and a higher elevation. Analogously, the right side of the pelvis in the no-LBP group also showed increased dorsal rotation, whereas in the LBP group, the left pelvis side exhibited dorsal rotation (Table 2).

**Table 2:** Comparison of the LBP group with the no-LBP group after Bonferroni-Holm correction

	No-LBP group		LBP group		*p*-value
	mean/median	SD/IQR	mean/median	SD/IQR	
Frontal trunk decline [°] *	-0.190	1.302	-0.7	1.269	< 0.001
Scapular rotation [°] *	1.29	4.38	-0.24	4.08	< 0.001
Scapular angle right [°] **	28.33	8.08	29.18	11.925	0.042
Pelvis rotation [°] **	0.51	4.93	-0.73	4.51	< 0.001

* normally distributed values: two-sample t-test, mean, SD; ** non-normally distributed values: Mann-Whitney-U-test, median, IQR

Similar comparisons of the females and males showed different results. While in the females most deviations seemed to happen in the scapular region (scapular rotation and scapular angle right) (Table 3), differences in the males with LBP occurred mainly in the frontal plane (Table 4). However, both sexes showed significant differences in the pelvis rotation which confirms the trends observed in the sex independent analysis (Table 2).

**Table 3:** Comparison of the LBP group with the no-LBP group in females after Bonferroni-Holm correction

+ dorsal - ventral	No-LBP group		LBP group		*p*-value
	mean/median	SD/IQR	mean/median	SD/IQR	
Standard deviation of rotation [°] **	4.18	8.883	3.47	7.308	0.044
Scapular rotation [°] **	1.75	4.64	-0.19	4.11	< 0.001
Scapular angle right [°] **	28.49	9.26	31.46	18.588	0.028
Pelvis rotation [°] **	1.18	11.828	-0.13	4.272	0.004

* normally distributed values: two-sample t-test, mean, SD; ** non-normally distributed values: Mann-Whitney-U-test, median, IQR

**Table 4:** Comparison of the LBP group with no-LBP group in males after Bonferroni-Holm correction

	no-LBP group		LBP group		*p*-value
	mean/median	SD/IQR	mean/median	SD/IQR	
Frontal trunk decline [°] **	-0.16	1.66	-0.99	1.985	< 0.001
Pelvis rotation [°] **	-0.14	5.21	-2.27	4.165	0.004

* normally distributed values: two-sample t-test, mean, SD; ** non-normally distributed values: Mann-Whitney-U-test, median, IQR

### Regression analysis

In the regression analysis, the sagittal parameters (sagittal trunk decline, thoracic bending angle, lumbar bending angle, kyphosis angle and lordosis angle) were predominantly significant for the spine parameters in the dependent variables (Table 5). These parameters depended differently on gender. There were also dependencies on age, BMI or height and/or weight, but not of group membership; the latter correlated only with frontal trunk decline.

**Table 5:** Regression analysis

Dependent variable	Independent variable	Regression coefficient	Standard error	*p*-value
**Spine parameters**				
Sagittal trunk decline *	sex	1.341	0.248	< 0.001
	height	-8.346	3.999	0.037
	BMI	-0.384	0.129	0.003
Frontal trunk decline *	group	-0.504	0.121	< 0.001
Thoracic bending angle **	age	0.001	0.000	0.039
	sex	0.050	0.011	< 0.001
Lumbar bending angle **	sex	-0.115	0.012	< 0.001
	BMI	0.016	0.006	0.008
Standard deviation of rotation **	sex	-0.071	0.020	< 0.001
Kyphosis angle *	age	0.119	0.034	< 0.001
	sex	-8.524	1.159	0.001
	height	61.141	18.686	< 0.001
	weight	-0.742	0.201	< 0.001
	BMI	3.3226	0.602	< 0.001
Lordosis angle *	sex	-16.109	1.174	< 0.001
	BMI	1.714	0.610	0.005
**Shoulder parameters**				
Scapular rotation *	group	-1.213	0.305	< 0.001
**Pelvis parameters**				
Pelvis torsion *	height	-18.061	8.483	0.034
	weight	0.185	0.091	0.044
	BMI	-0.867	0.273	0.038
Pelvis rotation *	group	-1.489	0.338	< 0.001
	age	-0.022	0.010	0.032
	sex	-1.253	0.342	< 0.001

Asterisks indicate the type of regression performed: * linear regression; ** linear regression after transformation of the dependent variables

In the shoulder region, group membership also depended significantly on scapular rotation.

In the pelvic region, significant dependencies were only found in the transverse plane, namely between pelvic torsion and BMI, weight and height, as well as between pelvic rotation and group, age and sex.

## Discussion

The focus of the present analysis was on the comparison of overall upper body posture between people with and without low back pain, taking particular account of their gender, age and BMI. For this purpose, video raster stereography was used to evaluate not only the individual angles but also the entire spine, shoulder and pelvic area in the sagittal, frontal and transverse planes. Constitutional parameters such as the trunk length or shoulder and pelvic distance were excluded from the analysis.

Although the group comparison of all test subjects showed isolated significant differences in the frontal plane in the spine and shoulder areas, and in the transverse plane in the shoulder and pelvic areas, these were found to be not clinically relevant. These differences are predominantly within the range of measurement error or are very marginally outside of it, therefore, this observation appears to be a natural variance in posture. Similar results and conclusions can be seen in the gender-specific analysis. While the women predominantly showed significant rotational differences in all three areas (standard deviation of rotation, shoulder and pelvis rotation), the male participants only showed significance in the frontal trunk decline (frontal plane) and in the pelvis rotation (transverse plane).

Based on the available data, the previous findings of reviews by Chun et al. [12] and Chaléat-Valayer et al. [13] which showed that people with LBP tend to have a smaller lumbar lordotic angle (LLA) compared to age-matched healthy individuals could not be confirmed in the present study. In addition, our study could not confirm that there was a slight difference in the sagittal alignment of the spine and pelvis between patients with chronic low back pain and the control subjects. There were significant, albeit small, differences in the transverse and frontal planes but not in the sagittal plane. However, since the majority of CT and MRI scans are obtained with the patient in a supine position, and X-rays as well as whole spine scans are conducted while the patient is standing, including back scans, comparing the results of sagittal spine alignment using different methods may lead to misleading interpretations [22, 23].

The subsequent regression analysis analysed the influence of the independent variables group (LPB/no-LBP), age, BMI, weight, height and sex (male/female) with regard to the evaluation parameters for each area. Here, the independent variable group showed an effect on only a few dependent variable correlations (frontal trunk decline, scapular rotation and pelvis rotation). Thereby, the variables of sex and age dominated in the parameters of the sagittal plane. Other influencing factors such as age, BMI, height or weight also seem to have a significant influence on upper body posture more frequently than group affiliation. Age is mostly associated with gender and not with the group membership. Overall, sex appears to be the main influencing factor for upper body posture. Taking into account the regression coefficient and the clinical classification of this, a clinically meaningful consequence in relation to gender is only obtained for the sagittal parameters, i.e. the kyphosis and lordosis angles. Accordingly, the kyphosis angle was found to be 8.52° and the lordosis angle 16.11° lower in men than in women. With regard to the kyphosis angle, the BMI can also be classified as a relevant influencing factor. f the BMI increased by a value of 1, then the kyphosis angle was found to increase by 3.32°. As the BMI is calculated from the height and weight, these significant *p*-values can be classified as follows: if the height increases by 10 cm, then the kyphosis angle increases by 6.14° and if the weight increases by 1 kg, then angle decreases by 0.74°. Furthermore, in the sagittal plane, the aforementioned angles are the only clinically relevant results in the spinal region.

Previous analyses of the data from the no-LBP group by Ohlendorf et al. [20] yielded similar results as both the lordosis and kyphosis angles increased significantly with age regardless of gender. Women generally have higher values than men, being approximately 18° for the lordosis and 6° for the kyphosis angles [20]. Abrisham et al. [19] also found in 403 EOS imaging data that the mean lordosis angle was greater in women than in men in all age groups. Murrie et al. [24] also found no difference in lumbar lordosis between 27 LBP and 29 no-LBP subjects using magnetic resonance imaging (MRI). However, they also confirmed that lumbar lordosis is significantly more pronounced in women and people with a higher body mass index. Nonetheless, they were unable to detect any significant age-related changes. They attributed this to the genetic aspect that the female pelvis differs anatomically from the male. Mirzashahi et al. [15] also investigated the role of spinopelvic parameters as risk factors for non-specific lumbar spine pain using radiographic images. The comparison of 148 LBP and 148 controls showed that the female sex, higher BMI, smoking and blue-collar jobs were associated with a higher risk of non-specific LBP, whereby lumbar lordosis, sacral slope and pelvic tilt were all greater in LPB patients [15].

The positive correlations between BMI and the kyphosis angle have also been demonstrated in other studies. Increased body weight results in increased body mass; this creates a caudally directed load under the influence of gravity and, thus, favours a forward leaning posture. The kyphotic posture is reinforced by the anterior tilt of the body, shifting the body’s centre of gravity anteriorly [6, 25]. In this thematic context, Do Nascimento et al. [26] studied 25 volunteers (20w/5m) aged 18 to 40 years, 10 of whom had a BMI of 18 to 25 kg/m² and 15 of whom had a BMI ≤ 30 kg/m². Using visual inspection and the Balance System (Biodex), the overweight volunteers were found to have increased protrusion of the head, hyperkyphosis of the thoracic spine and hyperlordosis of the lumbar spine.

According to Souza et al. [27], the abdomen is displaced forward in overweight people which leads to an anterior shift of the body’s centre of gravity, resulting in increased lordosis and anteversion of the pelvis. The thoracic kyphosis also increases in this context. Increased body weight primarily affects the spine as it acts as a scaffold against gravity in an attempt to hold the person upright. The increased axial load caused by the increased body mass leads to attempts by the spine to compensate by adopting kyphotic, lordotic or scoliotic postures [26]. This results in overloading of the bones and joints and overstretching of the ligaments [28]. However, this could only be partially proven in the study data presented here as the lordosis angle was not significantly subject to this influence.

Limitations must also be taken into account in this study. When measuring people with a higher BMI, skin displacements or a thicker dermis, as found in obese patients, must be taken into account as these can lead to inaccuracies in the measurements. However, Drerup et al. [29], describe that the reproducibility of the marking of the vertebra prominens and the lumbar dimples (DL/DR) is 1 mm. The sensitivity of this method is 98% and the specificity 84% according to Asamoah et al. [30] compared to X-ray.

In order to be able to draw more precise conclusions and better comparisons with the back parameters, X-ray images should be included in future analyses. These would allow a better overview to be formed of the skeletal condition of the patient and, if necessary, information about any prolapse present. It would also be desirable to record the success of the therapy, i.e. data from the back scan as part of the therapeutic treatment, in order to gain a better understanding of the significance of pain or discomfort and its relation to the body’s posture. Pain sensitivity is an important issue that can severely affect a patient’s physical and mental state. It is, therefore, all the more important to pay close attention to this topic and to investigate it in the future.

## Conclusion

Previous studies have not investigated the thoracic as well as the frontal and transverse changes in upper body posture (spinal region with pelvic and shoulder area) due to LBP; the focus in previous studies has been mainly on the lumbar spine area from a sagittal view. In this study, an overall investigation of the key upper body measurements was employed and revealed no clinical relevant difference between healthy and LBP. In general, gender emerges as the primary factor influencing upper body posture. Other factors such as BMI, height, or weight also demonstrate a more consistent and substantial impact on upper body posture than group membership (LBP vs. no-LBP).

## Data Availability

No datasets were generated or analysed during the current study.

## Abbreviations

LBP: Low back pain
LSS: Lumbar spine syndrome
BMI: Body mass index
LLA: Lumbar lordotic angle
MRI: Magnetic resonance imaging
CT: Computed tomography
ROM: Range of motion
EOS: Low-dose X-ray device
PSIS: Posterior superior iliac spine
